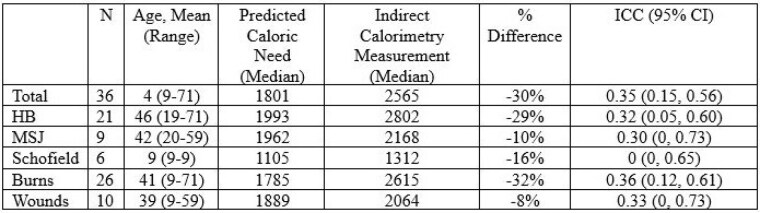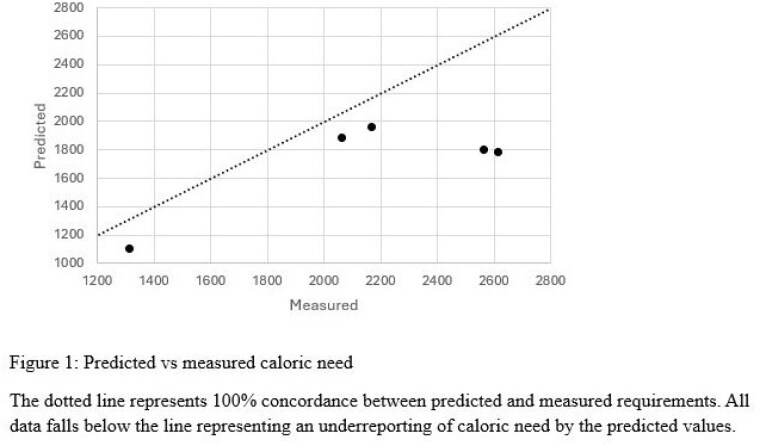# 79 Standardized Equations Underestimate Nutrition Needs in Burn and Complex Wounds Patients

**DOI:** 10.1093/jbcr/iraf019.079

**Published:** 2025-04-01

**Authors:** Brooke Hartzell

**Affiliations:** Regions Hospital Burn Center

## Abstract

**Introduction:**

Burns cause significant derangements in response to injury, leading to a prolonged hypermetabolism. The American Society of Enteral and Parenteral Nutrition (ASPEN) and burn literature recommend calorimetry (IC) as the gold standard for assessing nutrition needs. In the absence of IC, predictive equations are recommended. The use of standardized equations often does not capture the hypermetabolic state of burn injury and complex wounds. Appropriate nutrition support in the burn patient population is associated with lower incidence of complications, including pneumonia and sepsis, and reduced muscle protein catabolism. Using standardized equations in burn and complex wound patients fed enterally may underestimate the hypermetabolic demands in comparison to measured energy expenditure via IC.

**Methods:**

We conducted a retrospective observational study of the IC measurements conducted at our institution’s burn center and compared the results to the traditional method of predicting caloric requirements as determined by an equation (Harris Benedict (HB), Schofield, Mifflin-St Jeor (MSJ)). Thirty-six IC measurements were conducted during the 17-month study period on 21 unique patients aged 9 to 71 years. Each measurement pair consisted of a predicted daily caloric need as determined by the appropriate predictive equation and measured caloric need determined by IC. To assess concordance, Intraclass correlation coefficient (ICC) estimates and their 95% confidence intervals were calculated based on ICC3 single fixed rater, two-way mixed effects model. Negative values for the lower bounds of the 95% CI for ICC were adjusted to zero to reflect the meaningful range of ICC.

**Results:**

When examining the total group measurements, the ICC estimation was 0.35 95% CI (0.15, 0.56). Predictive equations underreported caloric need by 30% compared to IC measurements. When analyzing based on equations, the HB group had an ICC estimation of 0.32 (0.05, 0.60) and underreported caloric need by 29%. The MSJ group had an ICC of 0.30 (0, 0.73), and the Schofield group had an ICC of 0 (0, 0.65). Subgroup analysis by injury class found this relationship consistent with the Burn group underreporting caloric need by 32% and the Wound group underreporting caloric need by 8%, with ICC estimations of 0.36 95% CI (0.12, 0.61) and 0.33 (0, 0.73) respectively.

**Conclusions:**

Our data demonstrates that use of IC better represents the hypermetabolic response to burns and complex skin injury in this patient population in comparison to standardized equations.

**Applicability of Research to Practice:**

This study demonstrates that the use of IC more accurately reflects hypermetabolism in burn and complex wound patients and should continue to be the recommended method for estimating nutrition needs.

**Funding for the Study:**

N/A